# Valve-in-valve transcatheter aortic valve replacement (TAVR) leads to lower device success compared to TAVR in native stenosis

**DOI:** 10.3389/fcvm.2025.1465409

**Published:** 2025-04-22

**Authors:** Michael Paukovitsch, Bartu Dilaver, Dominik Felbel, Marvin Krohn-Grimberghe, Dominik Buckert, Johannes Moerike, Leonhard Moritz Schneider, Christian Liewald, Wolfgang Rottbauer, Birgid Gonska

**Affiliations:** ^1^ Department of Cardiology, Ulm University Heart Center, Ulm, Germany; ^2^ Department of Cardiothoracic and Vascular Surgery, Ulm University Heart Center, Ulm, Germany

**Keywords:** valve in valve aortic replacement, bioprosthetic valve degeneration, transcatheter aortic replacement, device success, bioprosthetic valve failure

## Abstract

**Background:**

Despite the lack of randomized-controlled trials in patients with failed bioprosthetic valves, valve-in-valve transcatheter aortic valve replacement (ViV-TAVR) is increasingly used.

**Methods:**

Outcomes of consecutive patients treated with ViV-TAVR (*N* = 100) at our tertiary heart center between 2014 and 2022 were compared to TAVR (*N* = 2216) in native valves.

**Results:**

Patients median age was 78.5 years (IQR 70.0–84.0) in ViV-TAVR compared to 81.0 (IQR 77.0–85.0) in patients with native aortic stenosis (*p* < 0.01) with a similar percentage of females in both groups (42% vs. 49.3%, *p* = 0.18). The median Society of Thoracic Surgeons score for mortality was significantly higher in patients undergoing ViV-TAVR [5.1% {IQR 2.6%–8.6%} vs. 3.8% {IQR 2.4%–6.3%}, *p* < 0.01]. ViV-TAVR was performed in degenerated surgical bioprostheses in 88% and in degenerated transcatheter bioprostheses in 12%. Stenosis was the main mechanism of bioprosthetic valve failure (70%), whereas severe regurgitation was the leading cause in 30%. The overall rate of device success amounted to 66% in ViV-TAVR, compared to 96.1% in TAVR (*p* < 0.01) and ViV-TAVR was independently associated with reduced device success (OR: 0.07, 95%CI: 0.045–0.12, *p* < 0.01) in multivariate regression. While ViV-TAVR decreased peak and mean gradients significantly, in 31% of patients elevated mean gradients (≥20 mmHg) were observed at discharge. Small native prosthesis diameter (<20 mm) was the strongest predictor (OR 3.8, 95%CI: 1.5–9.2, *p* = 0.01) independently associated with elevated gradients after ViV-TAVR.

**Conclusion:**

ViV-TAVR for treatment of degenerated bioprostheses improves aortic valve function. However, device success is lower compared to TAVR in native aortic valve disease, mainly due to elevated postprocedural mean gradients, especially in small bioprostheses.

## Introduction

Given the limited durability of bioprosthetic surgical and transcatheter aortic valves many patients require reoperation or reintervention due to native bioprosthesis degeneration. Especially re-do surgery, which had been the former standard, is considered a high-risk procedure not suitable for most of these elderly patients (1, 2). Valve-in-valve transcatheter aortic valve replacement (ViV-TAVR) has been shown to be a safe alternative for treatment of bioprosthetic valve failure (BVF) in these patients (3, 4). While most TAVR procedures are still performed in native valves, the percentage of ViV-TAVR procedures is expected to rise as life-expectancy is increasing.

We evaluated short and mid-term outcomes of ViV-TAVR procedures performed at our high-volume tertiary heart center by comparing procedural outcome to TAVR in native valve stenosis.

## Methods

### Study population and procedural details

**Figure 1:**
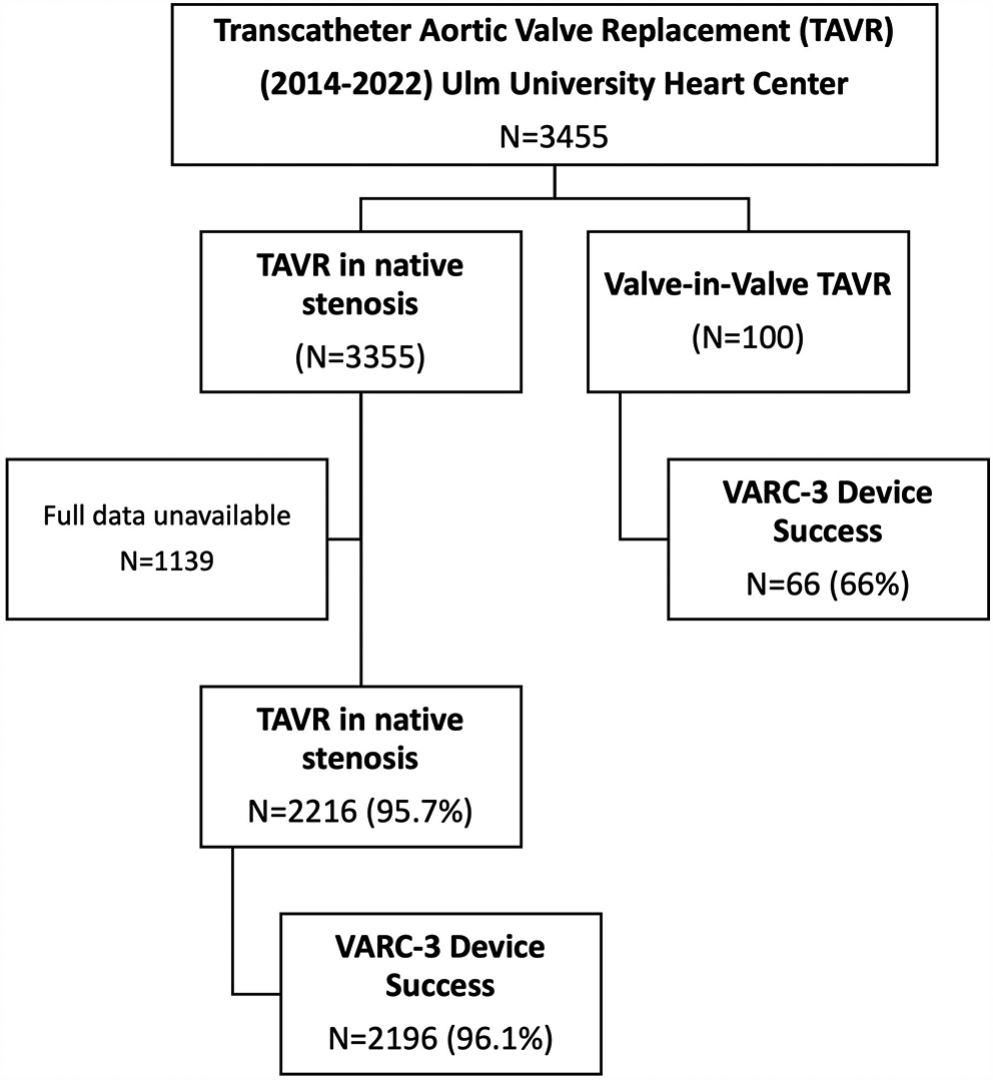
(Study flowchart): overall 3,455 were treated with TAVR at Ulm university heart center between 2014 and 2022. 100 patients were treated with ViV-TAVR. Data regarding device success was available in 2,216 patients treated with TAVR for native valve stenosis. These patients were used to compare device success to patients treated with ViV-TAVR.

Between January 2014 and December 2022 3,455 patients were treated with TAVR at our university heart center of whom 100 patients had ViV-TAVR procedures due to failed aortic bioprostheses (2.9% of all patients). For 2,216 out of 3,355 patients receiving TAVR in native valve stenosis sufficient retrospective data regarding interventional risk (STS Score for mortality) and in-hospital device success rate were available. These were used as a reference standard for comparison to ViV-TAVR (see Figure 1).

All patients were evaluated by the local heart team and directed towards ViV-TAVR based on the heart team's decision. TAVR procedures were guided using fluoroscopy.

Procedures were performed under local anesthesia and mild conscious sedation, if required. Transfemoral access was exclusively used in ViV-TAVR patients. Patients received standard single antiplatelet therapy, anticoagulation only if otherwise indicated.

All patients provided written informed consent for data collection and analysis (The authors confirm that patient consent forms have been obtained for this article). This study was approved by the local ethics committee (Ethics committee of Ulm University) and meets the standards set out in the Declaration of Helsinki.

Peak and mean gradients were measured using transthoracic echocardiography. Postprocedural gradients (before discharge) were available in all but two patients. One patient died, whereas another patient suffered a major stroke before documentation of postprocedural TTE could occur.

All patients underwent preprocedural 256 multislice contrast-enhanced computed tomography, which was evaluated with a dedicated software (3mensio Structural Heart 9.1 software, Pie Medical Imaging B.V., Maastricht, The Netherlands). CT evaluation included measurement of the true inner diameter and area of the prosthesis, measurements of the left-ventricular outflow tract, sinotubular junction, distance of the coronary arteries to the base of the prosthesis as well as of peripheral vessels according to a standardized protocol.

As previously described (5), the choice of valve to be implanted into the degenerated prosthesis was based on CT measurements (measured true inner diameter and area) as well as on the sizing app published by Bapat et al. (6).

30-day follow-up was scheduled for all patients in an out-patient capacity. If the patient did not show up for follow-up records from external physicians or telephone interview was used instead. In ViV-TAVr patients, 30-day and 1-year follow-up data was available for 95 patients and 86 patients, respectively. Echocardiography at 30-day and 1-year follow-up was available for 82 and 64 patients, respectively. In the propensity score matched analysis data on 30-day all-cause mortality was available for 248/288 (88.1%) of patients.

### Definitions

Bioprosthetic valve failure (BVF) was determined using a combination of clinical (new onset symptoms/worsening symptoms, LV dysfunction), morphological (wear and tear, leaflet disruption, leaflet fibrosis/and or calcification) as well as hemodynamic criteria (increased gradients in serial measurements) leading to reintervention (based on present day VARC-3 criteria) (7). The mechanism of BVF was assigned according to the leading cause of failure and patients were sorted into either the “stenosis” or “regurgitation” group.

The definition of device success adheres to the current VARC-3 criteria (7), which require a postprocedural mean echocardiographic gradient ≤20 mmHg and absence of greater or equal to moderate aortic regurgitation. Device success was analyzed at discharge (in-hospital device success). Technical success was defined according to the same recommendation (7) (successful device deployment, freedom from mortality, correct positioning of a single device, freedom from surgery/intervention related to the device).

### Statistical analysis

Continuous variables were analyzed for distribution graphically using histograms and Q-Q plots. Normally distributed variables were shown as mean ± standard deviation, whereas non-normally distributed variables were shown as median and interquartile range (IQR).

Nominal variables were shown as frequencies and percentages.

Statistical testing was performed using the Student t-test for normally distributed variables and the Mann–Whitney test for non-normally distributed variables, respectively. In case of paired variables testing was performed likewise, using the paired Student *t*-test and Wilcoxon test, respectively. Categorical variables were tested using the Chi-square or the Fisher's exact test, as appropriate.

For analysis of predictors of elevated gradients patients were first dichotomized according to the presence of elevated gradients (before discharge). Variables significantly differing between groups were further tested in univariate binary logistic regression and were shown as odds ratio and respective 95% confidence intervals. Multivariate logistic regression was not necessary as only one variable remained a significant predictor of elevated gradients in univariate logistic regression.

For analysis of device success, patients were grouped according to non-device success and device success. Variables significantly differing between groups were analyzed in univariate binary logistic regression. Significant variables were further tested in multivariate regression to adjust possible predictors of device success for covariates. Multicollinearity was tested using Pearson and Spearman's correlation coefficients (*r* ≤ 0.4) as well as variance inflation factor (VIF < 10).

In an additional analysis, 1:2 propensity score matching was conducted using baseline variables which differed significantly between patients with ViV-TAVR and those with native aortic stenosis (sex category, balloon vs. self-expandable valve, STS score for mortality, age, and AV mean and max gradients). Exact matching was used for the variables sex category and balloon expandable valve. Age, STS score, AV mean and max gradients were matched using an optimal matching approach without caliper restriction and replacement (8). A two-sided *p*-value of 0.05 was applied for all statistical testing. Statistical testing was performed using SPSS (SPSS, IBM Statistics), Version 29. Propensity score matching was conducted using SAS (verison 9.4).

## Results

### Overall cohort

**Figure 2:**
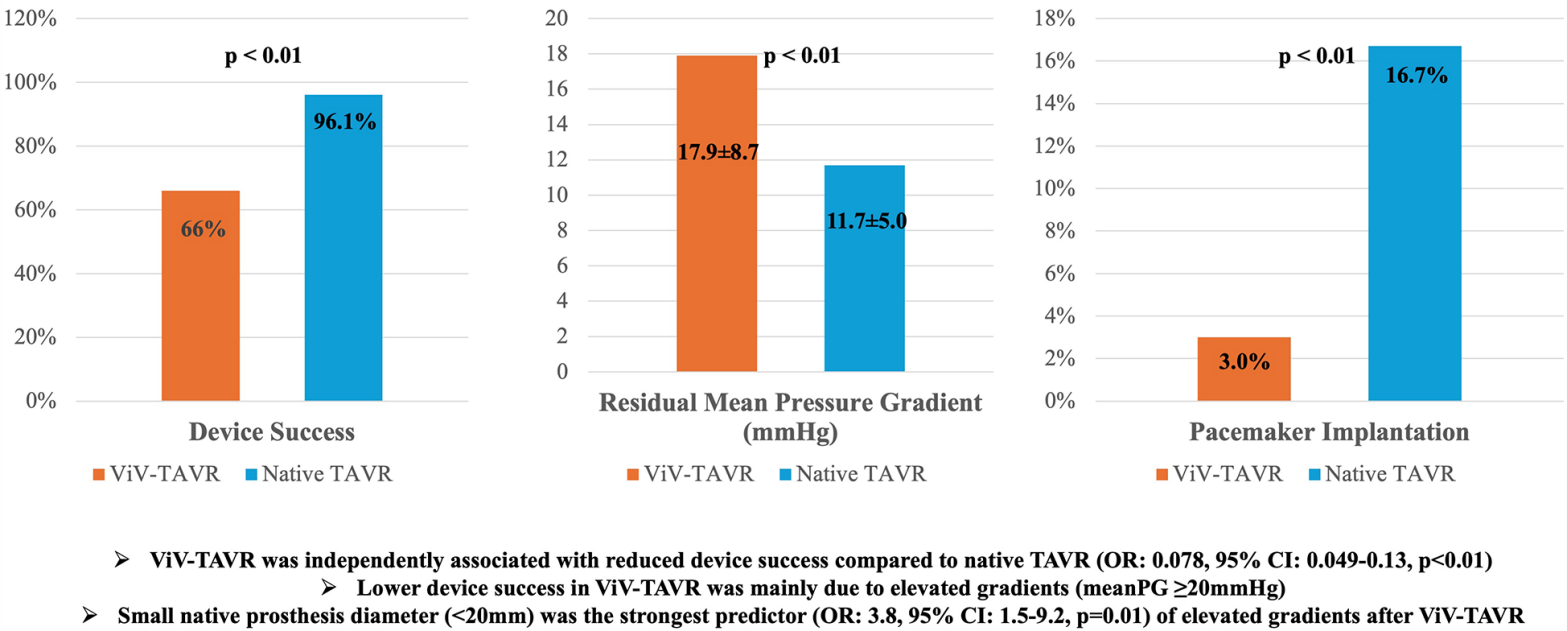
In-hospital outcomes of 2,316 patients treated with transcatheter aortic valve replacement (*N* = 2,216) or valve-in-valve (*N* = 100) transcatheter aortic valve replacement.

The overall study population included 2,316 patients who underwent ViV-TAVR (*N* = 100) or TAVR (*N* = 2,216) in native valve stenosis between 2014 and 2022 at the University Heart Center Ulm. Patients median age was 78.5 years (IQR 70.0–84.0) in ViV-TAVR compared to 81.0 (IQR 77.0–85.0) in patients with native aortic stenosis (*p* < 0.01). The percentage of females was similar in both groups [42 {42%} vs. 1,092 {49.3%}, *p* = 0.18]. Interventional risk according to the Society of Thoracic Surgeons score was significantly higher in patients undergoing ViV-TAVR [5.1% {IQR 2.6%–8.6%} vs. 3.8% {IQR 2.4%–6.3%}, *p* < 0.01] (see Table 1). Comorbidities such as diabetes mellitus [25 {25%} vs. 653 {29.5%}, *p* = 0.34], prior stroke [10 {10.0%) vs. 269 {12.1%}, *p* = 0.52] and coronary artery disease [65 {65.0%} vs. 1,374 {62.1}, *p* = 0.56] were similarly frequent in both patient groups. The central illustration (Figure 2) depicts in-hospital outcomes compared between ViV-TAVR and TAVR in native stenosis patients.

**Table 1:** Comparison of ViV-TAVR and TAVR in native aortic stenosis.

Parameter	Total *N* = 2,316	ViV-TAVR *N* = 100	Native TAVR *N* = 2,216	*p*
Age, years	81.0 (77–85.0)	78.5 (70.0–84.0)	81.0 (77.0–85.0)	**<0**.**01**
Female, *N* (%)	1,134 (49.0)	42 (42%)	1,092 (49.3)	0.18
BMI	27.1 ± 4.8	27.1 ± 4.8	26.9 ± 4.3	0.8
STS score risk for mortality, %	3.8 {2.4–6.4}	5.1 (IQR 2.6–8.6)	3.8 (2.4–6.3)	**<0**.**01**
AF, *N* (%)	888 (38.3)	42 (42.0)	846 (38.2)	0.44
AHT, *N* (%)	2,051 (88.6)	87 (87.0)	1,964 (88.7)	0.85
CAD	1,440 (62.3)	65 (65.0)	1,374 (62.1)	0.56
Diabetes mellitus	678 (29.3)	25 (25.0)	653 (29.5)	0.34
Prior stroke/TIA	279 (12.0)	10 (10.0)	269 (12.1)	0.52
Hb	12.3 ± 5.1	12.4 ± 1.9	12.3 ± 5.2	0.87
eGFR, ml/min	56.9 ± 24.5	56.9 ± 24.6	57.4 ± 23.0	0.84
AV mPG, mmHg	39.2 ± 15.4	36.0 ± 15.3	39.3 ± 15.4	**0**.**04**
AVmaxPG, mmHg	65.9 ± 23.8	61.5 ± 27.5	66.1 ± 23.6	**0**.**07**
NYHA III/IV	1,730 (74.7)	77 (77.0)	1,653 (74.6)	0.59
Balloon expandable valve (BEV)	1,106	36 (36.0)	1,070 (48.3)	**0**.**02**
Device success, *N* (%)	2,196 (94.8)	66 (66)	2,130 (96.1)	**<0**.**01**
Stroke, *N* (%)	61 (2.6)	1 (1.0)	60 (2.7)	0.29
Pacemaker implantation, *N* (%)	372 (16.1)	3 (3.0)	369 (16.7)	**<0**.**01**
In-hospital death	47 (2.0)	1 (2.0)	46 (2.1)	0.45
AV mPG post, mmHg	12.0 ± 5.4	17.9 ± 8.7	11.7 ± 5.0	**<0**.**01**
AVmaxPG post, mmHg	22.5 ± 9.9	32.2 ± 15.0	22.5 ± 9.9	**<0**.**01**

Values are shown as frequencies (*N*) and percentages (%), mean ± standard deviation (SD) or median and interquartile range (IQR).

Bold values indicate significant *p*-values.

AF, atrial fibrillation; AHT, arterial hypertension; AV, aortic valve; BMI, body mass index; BEV, Balloon-expandable valve; CAD, coronary artery disease; eGFR, estimated glomerular filtration rate; Hb, hemoglobin level; max PG, maximum pressure gradient; mPG, mean pressure gradient; NYHA, New York Heart Association; post, postprocedural; STS, Society of Thoracic Surgeons; ViV-TAVR, Valve-in-valve transcatheter aortic valve replacement.

### ViV-TAVR patients

88% received ViV-TAVR in degenerated surgical bioprostheses, 12% in degenerated transcatheter bioprostheses. The vast majority (77%) of patients was highly symptomatic with NYHA functional class III/IV class (further see Table 2). Carpentier Edwards (44%), Medtronic Freestyle (10%) and SJM trifecta (12%) were among the most frequent treated bioprosthetic valves. Overall, the majority of patients (86%) had been implanted with stented bioprosthetic valves.

**Table 2:** Surgical and transcatheter valve characteristics in ViV-TAVR patients.

Parameter	*N* (%)
Native bioprostheses type	
SAVR, *N* (%)	88 (88%)
TAVR, *N* (%)	12 (12%)
Stented, *N* (%)	86 (86%)
Stentless, *N* (%)	14 (14%)
Leading mode of native bioprostheses failure	
Stenosis, *N* (%)	70 (70%)
Regurgitation, *N* (%)	30 (30%)
Surgical valve/TAVR make and model	
Medtronic Freestyle, *N* (%)	10 (10%)
Carpentier Edwards, *N* (%)	44 (44%)
MitroFlow, *N* (%)	3 (3%)
SJM Trifecta, *N* (%)	12 (12%)
Sorin Solo Freedom, *N* (%)	1 (1%)
SJM Epic, *N* (%)	3 (3%)
Medtronic Hancock, *N* (%)	7 (7%)
Sorin Crown, *N* (%)	2 (2%)
SJM Toronto, *N* (%)	1 (1%)
Edwards Sapien XT, *N* (%)	4 (4%)
Lotus, *N* (%)	1 (1%)
Homograft, *N* (%)	1 (1%)
Edwards Sapien S3, *N* (%)	3 (3%)
Direct Flow, *N* (%)	4 (4%)
Magna Ease, *N* (%)	2 (2%)
Xenograft, *N* (%)	1 (1%)
Perceval L, *N* (%)	1 (1%)
Size group according to true inner diameter (ID)	
<20 mm, *N* (%)	38 (38%)
≥20 and <23 mm, *N* (%)	35 (35%)
≥23 mm, *N* (%)	27 (27%)

Values are shown as frequencies (*N*) and percentages (%).

SAVR, surgical aortic valve replacement; TAVR, transcatheter aortic valve replacement.

Stenosis was the leading mechanism of bioprosthetic valve failure in 70%, whereas severe regurgitation was the leading cause in 30%. A combined cause (stenosis with ≥II° aortic regurgitation) was observed in 32 patients. For the ViV procedure 62 patients were treated with a self-expandable Medtronic valve (CoreValve/Evolut), whereas 36 received the balloon-expandable Edwards Sapien 3 valve, 2 patients were treated with the mechanically expanded Boston Lotus Edge valve. 99% of patients had technical success. One patient died after successful device delivery going into cardiac arrest following hemorrhagic shock due to massive internal bleeding from the external iliac artery. There was no case of coronary obstruction. Temporary hemodynamic instability requiring inotropes was the most frequent intraprocedural complication (14%). Valve cracking (fracturing the ring of a degenerated bioprosthesis) was not performed in any of the patients.

Due to valve migration successful implantation of a second device was necessary in one patient.

99% had none/trace or mild postprocedural aortic regurgitation. 3% required postinterventional permanent pacemaker implantation.

### Device success

The overall rate of in-hospital device success amounted to 66% in ViV-TAVR (*N* = 66), compared to 96.1% in TAVR (*N* = 2,130) (*p* < 0.01) (see Table 3; Figure 2). Among ViV-TAVR patients non-device success was driven by elevated postprocedural mean gradients (≥20 mmHg) in the majority of cases (31%). Multivariate logistic regression found ViV-TAVR to be inversely associated with device success (OR: 0.07, 95% CI: 0.045–0.12, *p* < 0.01) after adjusting for covariates (see Supplementary Tables S1, S2). Moderate aortic regurgitation was present in another patient (1%). Two patients did not receive echocardiography before discharge (see also Methods section) and were also counted as non-device success. Among ViV-TAVR patients, postprocedural mean and peak gradients decreased significantly (both *p* < 0.01) to 17.9 ± 8.7 and 32.2 ± 15.0 mmHg, respectively.

**Table 3:** Echocardiography and procedural outcomes in ViV-TAVR patients.

Parameter	N (%)/Mean ± SD
Echocardiography	
LV EF, %	48.6 ± 13.4
Aortic regurgitation ≥ II, N (%)	32 (32%)
AV mPG, mmHg pre	36.0 ± 16.8
AV maxPG, mmHg pre	61.5 ± 27.5
AV mPG, mmHg post (*N* = 98)	17.9 ± 8.7
AV maxPG, mmHG post (*N* = 98)	32.2 ± 15.0
Elevated gradients (≥20 mmHg mPG)	31 (31%)
Type of implanted TAVR	
BEV (Sapien platform), *N* (%)	36 (36%)
SEV (Core Valve platform), *N* (%)	62 (62%)
Lotus, *N* (%)	2 (2%)
Device time (min)	58.5 (49.5–73.3)
Preimplantation valvuloplasty, *N* (%)	69 (69%)
Postimplantation valvuloplasty, *N* (%)	8 (%)
Postprocedural aortic regurgitation (≥2), *N* (%)	1 (1%)
Successful valve deployment, *N* (%)	100 (100%)
In-hospital outcome	
Technical success, *N* (%)	99 (99%)
In-hospital device success, *N* (%)	66 (66%)
In-hospital complications, *N* (%)	
Intra-procedural death, *N* (%)	1%
Pericardial effusion, *N* (%)	0
Stroke, *N* (%)	1%
Coronary Obstruction, *N* (%)	0
Conversion to open heart surgery, *N* (%)	0
Pacemaker implantation, *N* (%)	3 (3%)
30-day follow-up	
All-cause mortality, *N* (%)	4 (4.2%)
Valve-related, *N* (%)	1 (1.1%)
CV death, *N* (%)	3 (3.1)
Non-CV death, *N* (%)	0
Stroke, *N* (%)	1 (1.1%)
Myocardial Infarction, (*N*%)	0
Pacemaker implantation, (*N*%)	5 (5.2%)
mPG, mmHg (*N* = 82)	18.6 ± 8.8
*Δ* mPG (compared to discharge), mmHg	−0.9 ± 5.1
12-month follow-up	
All-cause mortality, *N* (%)	9 (10.4%)
Valve-related, *N* (%)	1 (1.2%)
CV death, *N* (%)	3 (3.4%)
Non-CV death, *N* (%)	5 (5.8%)
Stroke, *N* (%)	1 (1.2%)
Myocardial Infarction, *N* (%)	0
Pacemaker Implantation, *N* (%)	5 (5.8%)
mPG, mmHg (*N* = 64)	19.2 ± 10.0
*Δ* mPG (compared to discharge), mmHg	−0.2 ± 6.9

Values are shown as frequencies (*N*) and percentages (%), mean ± standard deviation (SD) or median and interquartile range (IQR).

AV, aortic valve; BEV, balloon expandable valve; CV, cardiovascular; LV EF, left-ventricular ejection fraction; mPG, mean pressure gradient; maxPG, maximum pressure gradient; SEV, self-expandable valve.

### Device success in the propensity score matched cohort

In a second analysis, 1:2 propensity score matching was conducted to account for differences in baseline variables between patients receiving ViV-TAVR and TAVR in native aortic stenosis. Matching was conducted for baseline variables differing significantly between these groups (see Table 1; Supplementary Table S2). A match was found for 96/100 patients with ViV-TAVR, thus 96 patients with ViV-TAVR were compared to 192 patients with TAVR in native stenosis. Variables were well balanced in the matched cohort (see Supplementary Table S2).

In the matched cohort, device success was also significantly lower in ViV-TAVR patients compared to those with TAVR in native stenosis (64.4 vs. 94.8%, *p* < 0.01). 30-day all-cause mortality (5.4% vs. 4.5%, *p* = 0.77) did not differ between ViV-TAVR and TAVR patients in the propensity score matched cohort.

### 30-day and 12-month clinical and safety outcomes in ViV-TAVR patients

All-cause mortality at 30 days was 4.2%, with one procedure-related and three CV deaths. Stroke occurred in one patient (during the initial clinical stay). Two additional patients required pacemaker implantation within 30 days after discharge. Mean transvalvular gradients remained stable compared from discharge to 30-day follow-up (−0.9 ± 5.1 mmHg, *p* = 0.12). At one-year follow-up overall all-cause mortality amounted to 10.4% (3.4% CV-deaths) with neither additional strokes, myocardial infarction nor pacemaker implantation occurring during the follow-up period. Mean transvalvular gradients remained stable compared from discharge to 30-day follow-up (−0.9 ± 5.1 mmHg, *p* = 0.12) and compared from discharge to 1-year follow-up (−0.2 ± 6.9, *p* = 0.8).

### Elevated gradients at discharge

Elevated mean valvular gradients exceeding 20mmHg were found in 31/98 patients (see also Table 4) with available echocardiography at discharge and amounted to 28.5 ± 6.6 vs. 12.9 ± 3.8 mmHg on average when comparing patients grouped according to elevated mean gradients (mPG > 20 mmHg). Patients with elevated gradients had exclusively received native bioprosthetic valves with a stented design. Bioprostheses with small internal diameter (<20 mm) were more frequent among these patients (58.1% vs. 26.9%, *p* < 0.01).

**Table 4:** Comparison of patients receiving ViV-TAVR with and without elevated gradients (≥20 mmHg) at discharge.

*N* = 98	Non-elevated gradients (*N* = 67)	Elevated gradients (*N* = 31)	*p*	
Age, years	79.0 (69.0–83.0)	81.0 (73.0–87.0)	0.26	
Female, *N* (%)	26 (38.8)	15 (48.4)	0.37	
STS score risk of mortality, %	4.8 (2.5–9.3)	5.2 (3.2–8.4)	0.69	
LV EF, %	46.9 ± 13.4	52.2 ± 13.0	0.08	
Aortic regurgitation ≥II	24 (35.8)	7 (22.6)	0.19	
AV mPG, mmHg pre	34.1 ± 16.8	40.0 ± 16.8	0.11	
AV maxPG, mmHg pre	58.1 ± 26.9	68.4 ± 26.3	0.09	
AV mPG, mmHg post	12.9 ± 3.8	28.5 ± 6.6	**<0**.**01**	
AV maxPG, mmHG post	24.2 ± 7.0	59.6 ± 12.7	**<0**.**01**	
Measured true internal diameter (average), mm	21.6 ± 3.2	19.9 ± 2.5	**0**.**01**	
Measured true internal diameter (average) size groups				
<20mm	18 (26.9)	18 (58.1)	**<0**.01*	**0**.01**
20–23mm	27 (40.3)	10 (32.3)		
>23mm	22 (32.8)	3 (9.7)		
BEV, *N* (%)	25 (37.3)	9 (29.0)	0.37	0.41
SEV, *N* (%)	40 (59.7)	22 (71.0)		
Lotus, *N* (%)	2 (2.9)	0		
Stentless native prosthesis design	13 (19.4)	0	**<0**.**01**	
Mode of failure (regurgitation)	23 (34.3)	6 (19.4)	0.13	
Balloon predilation	46 (68.7)	22 (71.0)	0.82	
Balloon postdilation	5 (7.5)	3 (9.7)	0.71	
Permanent Pacemaker Implantation	2 (3.0)	1 (3.2)	1.0	

Values are shown as frequencies (*N*) and percentages (%), mean ± standard deviation (SD) or median and interquartile range (IQR).

AV, aortic valve; BEV, balloon expandable valve; LV EF, left-ventricular ejection fraction; mPG, mean pressure gradient; maxPG, maximum pressure gradient; SEV, self-expandable valve; STS, Society of Thoracic Surgeons.

* This *p*-value refers to the chi-square test comparison in patients with diameters <20 mm.

** This *p*-value refers to the chi-square test comparison across all diameters groups.

In logistic regression analysis (Table 5), small (<20 mm) native prosthesis internal diameter (OR 3.8, 95% CI 1.5–9.5, *p* = 0.01) was independently associated with elevated gradients at discharge. Of note, as none of the patients with elevated gradients had a stentless prosthesis design, this parameter could not be included into the regression model. Among patients with elevated gradients (*N* = 31) 3 patients received a CT scan, whereas 6 patients received a TEE within 1 month after ViV-TAVR. Early thrombosis was found in 2 patients and oral anticoagulation was initiated. Elevated gradients were attributed to the ViV-TAVR procedure and went without consequence in the rest of these patients.

**Table 5:** Predictors of elevated (mPG ≥ 20 mmHg) gradients at discharge (logistic regression) in ViV-TAVR patients.

Univariate	OR (95% CI)	*p*
LV EF	1.03 (0.99–1.07)	0.08
AV mPG pre	1.02 (0.99–1.05)	0.12
AV maxPG pre	1.01 (0.99–1.03)	0.09
Measured (CT) true internal diameter (average)	0.82 (0.7–0.97)	**0**.**02**
Measured (CT) true internal diameter (average) <20 mm	3.8 (1.5–9.2)	**0**.**01**
Stentless prosthesis	n/a	

Variables are shown as odds ratio (OR) and 95% confidence interval. AV, aortic valve; LV EF, left ventricular ejection-fraction; max PG, maximum pressure gradient; mPG, mean pressure gradient.

Multivariate regression was not performed as only the measured true internal diameter remained significant.

## Discussion

### Safety profile and device success

Patients with bioprosthetic valve failure are increasingly treated with valve-in-valve TAVR instead of surgical reintervention due to prohibitive risk of re-do surgery. We retrospectively analyzed early outcomes of 100 patients treated with ViV-TAVR at our tertiary heart center. The device success rate and interventional risk according to the STS score of mortality of 2,216 patients undergoing TAVR in native aortic stenosis was used as a comparison for ViV-TAVR procedural outcomes: Interventional risk of ViV-TAVR is significantly higher [median STS score: 5.1% {2.6%–8.6%} vs. 3.8% (2.4%–6.3%), *p* < 0.01], whereas device success according to VARC-3 criteria is much lower compared to TAVR in native aortic stenosis (66% vs. 96.1%, *p* < 0.01). After adjusting for covariates potentially affecting device success, ViV-TAVR was independently associated with lower likelihood for device success (OR: 0.07, 95% CI: 0.045–0.12; *p* < 0.01). Moreover, when using propensity score matching device success was similarly significantly lower in ViV-TAVR patients (64.4 vs. 94.8%, *p* < 0.01).

Nevertheless, ViV-TAVR procedures had roughly doubled at our center between 2019 and 2022 compared to the years before on average.

Previous studies have shown high technical success with ViV-TAVR and an acceptable safety profile (3, 4, 9–11) with 30-day mortality ranging from 1.3% to 7.6% (4, 9–17). However, one further study with high-risk patients reported 11% 30-day mortality (18). Notably, 30-day mortality did not differ between ViV-TAVR and native TAVR patients (5.4% vs. 4.5%, *p* = 0.77) in our propensity score matched analysis. Moreover, re-do TAVR (TAVR in TAVR) was found to have a similar safety profile with a large registry reporting a 1.4% 30-day mortality in patients with native TAVR dysfunction beyond one year of its original implantation (10). Earlier registries such as the Valve-in-Valve International (VIVID) Registry (4) reported a higher mortality rate (7.6%). Decreasing mortality in ViV-TAVR patients is partly due to growing experience with the procedure as well as to the expansion of ViV-TAVR to intermediate and low-risk patients as opposed to a strategy of last resort for high risk, frail and elderly patients (19). Moreover, mortality may vary across studies depending on the heart team's experience and patients’ comorbidities.

While a 7.6% or higher 30-day mortality rate might be deemed unacceptable in the field of present day native TAVR, the growing body of evidence has consistently shown ViV-TAVR to be comparable to native TAVR regarding its safety profile (11, 16, 20). Moreover, outcomes have been shown to be more favorable compared to re-do surgery (21). The 30-day mortality rate of 4.2% observed in our study is thus within the rates previously observed and slightly lower than the expected mortality rate of 5.1% based on the median STS score. 12-month all-cause mortality in ViV-TAVR patients amounted to 10.4% in our study, which is likewise comparable to other ViV-TAVr registries with 12-month mortality ranging from 9.6%–12.4% (9, 12, 14, 15, 18). Moreover, this seems to be acceptable when compared to intermediate risk patients (STS score for mortality: ≥4% to ≤8%) undergoing TAVR in native stenosis, who have a similar risk compared to patients in our study with a median STS score of 5.1% {IQR: 2.6%–8.6%}: In randomized-controlled trials comparing TAVr to surgery (22, 23) 14.5% (22) and 12.6% (23) 12-month mortality rates were reported.

Large registries report stroke to occur within 1%–2.8% (4, 9–12, 17, 18) of patients, which seems to be comparable to the stable incidence of 2.3% (24) seen in native TAVR procedures. A cerebral protection device (CEP) was used in 64% of patients in our study, and according to a recent randomized-controlled trial the impact of CEP remains undetermined (25), however, we observed a very low 1% incidence of stroke in our cohort.

Fewer patients require permanent pacemaker implantation after ViV-TAVR compared to native TAVR, as the native prosthesis ring supposedly provides protection for the conduction system (5, 26). Hence, very few (3%) of patients required pacemaker implantation during their clinical stay in our study.

Significant (≥moderate) aortic regurgitation was only present in one patient, therefore having little impact on VARC-3 defined device success. However, elevated postprocedural transvalvular gradients were frequently observed in our study, especially in patients with stenosis as the leading cause of BVF. Consecutively, in-hospital device success was lower compared to native TAVR procedures mainly due to a higher percentage of small native bioprostheses in these patients compared to those with device success.

Many registries such as the VIVID (Global Valve in Valve Registry) (3, 4) also reported relatively low device success rates (58.9%) foremost attributable to elevated gradients (3). Further studies (9, 13, 18, 20), including such directly comparing ViV- and native aortic valve replacement (12), frequently see elevated gradients in ViV-TAVR patients likewise.

### Prosthesis size determines postprocedural gradients

Previous studies (4, 9) as well as our study found smaller native internal prosthesis diameters in patients with elevated gradients, which independently predicted elevated gradients in our cohort (OR: 3.8, 95% CI 1.5–9.2, *p* = 0.01). Furthermore, none of the patients with stentless native prosthesis design, which can be expanded more easily during ViV-TAVR, had elevated gradients. Nevertheless, in patients with stentless prosthesis design the predominant mode of failure prosthesis was severe regurgitation.

Some studies determined elevated gradients to negatively effect both quality of life (15) and mortality (4, 9), whereas others (12, 15) have found no negative association between elevated gradients and mortality. It has been previously pointed out, that VARC criteria for device success were developed for native TAVR (3), which might thus be not suitable for ViV-TAVR.

Theoretically, small bioprosthestic valves can be enlarged by fracturing the bioprosthesis’ ring using a non-compliant balloon (27), also known as “valve cracking.” Valve cracking has an acceptable safety profile and leads to lower transvalvular gradients (28, 29). The long-term benefit of valve cracking is not ultimately determined, especially when taking into account the increase in procedural risk: Both perforation and coronary obstruction due to narrowing of the virtual transcatheter heart valve to coronary (VTC) distance (28) may complicate valve cracking. Additional studies providing more evidence on improved long-term outcome with vs. without valve cracking are desirable. At last, valve cracking is only feasible in some valves and considered impossible in valves with a hard ring structure such as the Hancock or Trifecta valves, which were used in 7 (3 Hancock, 4 Trifecta) out of 31 patients with elevated gradients in our study, respectively. While valve cracking was not performed in any of the patients in this cohort, this might have potentially led to lower gradients in ViV-TAVR patients. However, valve cracking was not common practice in the earlier days of ViV-TAVR, which includes many of the patients included in this study. Valve cracking was considered in later patients in this cohort, but not performed for anatomical (narrow VTCs and risk of coronary obstruction) or technical reasons (non-crackable valves). Although valve-cracking was not investigated in this study, it may be considered to achieve lower gradients in ViV-TAVR patients, but needs to be weighed against the increase in procedural risk. Moreover, postdilatation can reduce gradients up to a certain degree. Use of postdilatation was initially low in this cohort, but increased towards the end of this study's enrollment period.

## Limitations

We presented the findings from a single-center retrospective registry. Clinical as well as echocardiographic follow-up data was not available for all patients (see also Methods section). All procedures were performed by experts in a highly standardized manner after careful preprocedural planning. Retrospectively the number of patients deemed ineligible for a ViV-TAVR procedure (e.g., due to anatomical reasons) is undeterminable. While this most likely reflects real-world practice, any influence on outcome and results can therefore not be ultimately ruled out. Valve cracking was not performed in patients presented in this study. Valve cracking might potentially increase the rate of device success seen in ViV-TAVR patients, although this confers a higher interventional risk, which may also affect device success.

Device success was determined using echocardiographic gradients, which might differ from invasively determined gradients. Moreover, device success is a parameter developed for TAVR in native stenosis, which complicates its use in ViV-TAVR.

## Conclusion

ViV-TAVR improves aortic valve function and can be performed with an adequate short-term safety profile at a large tertiary center with long-standing experience in native TAVR procedures. However, device success is lower compared to TAVR in native aortic valve disease, mainly due to elevated postprocedural mean gradients in small bioprostheses. Lower device success in ViV-TAVR does not seem to negatively affect short term outcome when compared to TAVR in native stenosis.

## Data Availability

The original contributions presented in the study are included in the article/Supplementary Material, further inquiries can be directed to the corresponding author.
