# Supplementary material for: Valve-in-valve transcatheter aortic valve replacement (TAVR) leads to lower device success compared to TAVR in native stenosis
Source: Front Cardiovasc Med. 2025 Apr 22;12:1465409. doi: 10.3389/fcvm.2025.1465409 (PMC12086335; doi:10.3389/fcvm.2025.1465409)
Supplement: Supplementary file 1 [file Datasheet1.pdf]

Supplementary Table 1 – Comparison baseline characteristics of ViV-TAVR and TAVR in native aortic stenosis				
	**Total N=2316**	**Non-Device Success N=120**	**Device Success N=2196**	**p**
Age, years	81.0 {77.0-85.0}	79.0 {75.0-85.0}	81.0 {77.0-85.0}	**<0.01**
Female, N (%)	1134 (49.0)	67 (55.8)	1067 (48.6)	0.12
BMI	27.1±4.8	27.8±4.8	27.0±4.8	0.08
STS Score Risk for Mortality, %	3.8 {2.4-6.4}	3.9 {2.5-6.7}	2.4 {2.4-6.4}	0.56
AF, N (%)	888 (38.3)	40 (33.3)	848 (38.6)	0.25
AHT, N (%)	2051 (88.6)	102 (85.0)	1949 (88.8)	0.42
CAD	1440 (62.3)	71 (59.2)	1369 (62.4)	0.47
Diabetes mellitus	678 (29.3)	28 (23.3)	650 (29.6)	0.14
Prior stroke/TIA	279 (12.0)	12 (10.0)	267 (12.2)	0.48
Hb	12.3±5.1	12.4±1.8	12.3±5.2	0.76
eGFR, ml/min	56.9±24.5	58.0±22.1	56.8±24.7	0.63
AV mPG, mmHG	39.2±15.4	42.2±19.1	39.0±15.2	**0.01**
AVmaxPG, mmHg	65.9±23.8	71.2±28.7	65.6±23.5	**0.03**
NYHA III/IV	1730 (74.7)	89 (74.2)	1641 (74.7)	0.89
ViV-TAVR	100 (4.3)	34 (28.3)	66 (3.0)	**<0.01**
BEV, N (%)	1106 (47.8)	52 (43.3)	1054 (48.0)	0.32

Values are shown as frequencies (N) and percentages (%), mean ± standard deviation (SD) or median and interquartile range (IQR)

Bold values indicate significant p-values.

Abbreviations: AF = atrial fibrillation, AHT = arterial hypertension, AV = aortic valve, BEV = balloon-expandable valve, BMI = body mass index, CAD = coronary artery disease, eGFR = estimated glomerular filtration rate, Hb = hemoglobin level, max PG = maximum pressure gradient, mPG= mean pressure gradient, NYHA = New York Heart Association, STS = Society of Thoracic Surgeons, ViV-TAVR = Valve-in-valve transcatheter aortic valve replacement

Supplementary Table 2- Univariate und multivariate binary logistic regression analysis							
	**Univariate**				**Multivariate**		
	**OR**	**95%CI**	**p**		**OR**	**95%CI**	**p**
Age, years	1.04	1.02-1.07	**0.01**		1.01	0.99-1.046	0.18
BMI	0.97	0.94-1.00	0.08				
AV mPG, mmHG	0.99	0.98-0.99	**0.03**		0.98	0.97-0.995	**0.01**
AV maxPG, mmHg	0.99	0.98-0.99	0.14				
ViV-TAVR	0.078	0.049-0.13	**<0.01**		0.07	0.045-0.12	**<0.01**

Values are shown as odds ratio (OR) and its respective 95% confidence interval (CI).
Bold values indicate significant p-values.
The multivariate model included variables significant in univariate regression. maxPG was not included in the multivariate model due to autocorrelation with meanPG.
Abbreviations: AV = aortic valve, max PG = maximum pressure gradient, mPG= mean pressure gradient

Supplementary Table 3- Propensity Score Matched Cohort				
	**Total N=288**	**ViV-TAVR N=96**	**Native TAVR N=192**	**p-Value**
Device Success, N (%)	244 (84.7)	62 (64.6)	182 (94.8)	**<0.01**
Female, N (%)	126 (43.8)	42 (43.8)	84 (43.8)	1.0
Balloon-Expandable Valve, N (%)	102 (35.4)	68 (35.4)	34 (35.4)	1.0
STS Score	4.2 {2.4-8.3}	5.1 {2.6-8.5}	3.9 {2.3-8.0}	0.13
Age, years	77.0 {71.0-83.0}	78.0 {70.0-83.4}	77.0 {73.0-83.0}	0.61
AV meanPG, mmHg	35.8±15.5	36.0±16.8	35.7±14.8	0.87
AV maxPG, mmHg	61.5±24.6	62.2±27.3	61.1±23.2	0.72

Values are shown as frequencies (N) and percentages (%), mean ± standard deviation (SD) or median and interquartile range (IQR)
Bold values indicate significant p-values.
1:2 propensity score matching was conducted (ViV-TAVR vs. TAVR in native stenosis) using the variables which differed significantly between ViV-TAVR and TAVR in native stenosis patients (see Table 1).
Abbreviations: mPG= mean pressure gradient, max PG = maximum pressure gradient
